# Characterization of Influenza A and B Viruses Circulating in Southern China During the 2017–2018 Season

**DOI:** 10.3389/fmicb.2020.01079

**Published:** 2020-05-29

**Authors:** Yuqian Yan, Junxian Ou, Shan Zhao, Kui Ma, Wendong Lan, Wenyi Guan, Xiaowei Wu, Jing Zhang, Bao Zhang, Wei Zhao, Chengsong Wan, Weifeng Shi, Jianguo Wu, Donald Seto, Zhiwu Yu, Qiwei Zhang

**Affiliations:** ^1^Guangdong Provincial Key Laboratory of Tropical Disease Research, School of Public Health, Southern Medical University, Guangzhou, China; ^2^Guangdong Provincial Key Laboratory of Virology, Institute of Medical Microbiology, Jinan University, Guangzhou, China; ^3^Key Laboratory of Etiology and Epidemiology of Emerging Infectious Diseases in Universities of Shandong, Shandong First Medical University & Shandong Academy of Medical Sciences, Taian, China; ^4^Bioinformatics and Computational Biology Program, School of Systems Biology, George Mason University, Manassas, VA, United States; ^5^Division of Laboratory Science, The Affiliated Cancer Hospital & Institute of Guangzhou Medical University, Guangzhou, China

**Keywords:** influenza B virus, Yamagata lineage, hemagglutinin, Victoria lineage, phylogenetic analysis, mutation analysis, 3D structure analysis, antigenic sites

## Abstract

The trivalent seasonal influenza vaccine was the only approved and available vaccine during the 2016–2018 influenza seasons. It did not include the B/Yamagata strain. In this study, we report an acute respiratory disease outbreak associated with influenza B/Yamagata infections in Guangzhou, Southern China (January through March, 2018). Among the 9914 patients, 2241 (22.6%) were positive for the influenza B virus, with only 312 (3.1%) positive for the influenza A virus. The influenza B/Yamagata lineage dominated during this period in Southern China. The highest incidence of influenza A virus infection occurred in the children aged 5–14 years. In contrast, populations across all age groups were susceptible to the influenza B virus. Phylogenetic, mutations, and 3D structure analyses of hemagglutinin (HA) genes were performed to assess the vaccine-virus relatedness. The recommended A/H1N1 vaccine strain (A/Michigan/45/2015) during both 2017–2018 and 2018–2019 was antigen-specific for these circulating isolates (clade 6B.1) in Spring 2018. An outbreak of influenza B/Yamagata (clade 3) infections in 2018 occurred during the absence of the corresponding vaccine during 2016–2018. The recommended influenza B/Yamagata vaccine strain (B/Phuket/3073/2013) for the following season (2018–2019) was antigen-specific. Although there were only a few influenza B/Victoria infections in Spring 2018, five amino acid mutations were identified in the HA antigenic sites of the 19 B/Victoria isolates (clade 1A), when compared with the 2016–2018 B/Victoria vaccine strain. The number was larger than expected and suggested that the influenza B HA gene may be more variable than previously thought. One of the mutations (K180N) was noted to likely alter the epitope and to potentially affect the viral antigenicity. Seven mutations were also identified in the HA antigenic sites of 2018–2020 B/Victoria vaccine strain, of which some or all may reduce immunogenicity and the protective efficacy of the vaccine, perhaps leading to more outbreaks in subsequent seasons. The combined epidemiological, phylogenetic, mutations, and 3D structural analyses of the HA genes of influenza strains reported here contribute to the understanding and evaluation of how HA mutations affect vaccine efficacy, as well as to providing important data for screening and selecting more specific, appropriate, and effective influenza vaccine candidate strains.

## Introduction

Influenza viruses are the major cause of acute respiratory diseases in humans, causing several serious global pandemics because of their transmission dynamics and great antigenic variability. Compounding this is the potential of emergent strains originating in swine and avian hosts (Mostafa et al., 2018), as well as from reverse zoonosis from human to animals (Morens et al., 2013; Nelson and Vincent, 2015). For both directions of transmission, knowing the distribution of strains in human populations is important for perhaps predicting strains for the coming season.

Influenza A virus presents as a permutation of 18 hemagglutinin (HA) and 11 neuraminidase (NA) possible subtypes, with a wide range of hosts (Cauldwell et al., 2014). The latest two subtypes H17N10 and H18N11 are of bat origins (Tong et al., 2012, 2013). Influenza B virus, including the Victoria and Yamagata lineages, is hosted by humans and seals (Osterhaus et al., 2000). Although there is no direct evidence that influenza B is transmitted between humans and animals, it has been observed that influenza B can infect seal, swine, ferret, guinea pig, pheasant, dog and horse (Osterhaus et al., 2000; Pica et al., 2012; Huang et al., 2014; Ran et al., 2015; Pascua et al., 2016). Pandemic influenza is usually caused by influenza A virus, due to its rapid antigenic variation, strong replication capacity, and transmission ability associated with genetic reassortment (Chan et al., 2010; White and Lowen, 2018). Influenza B virus, often neglected, has been circulating and, in some seasons, has predominated over influenza A, particularly among children (Paul Glezen et al., 2013; Tewawong et al., 2015; Furuse et al., 2016). Vaccines against influenza A/H1N1 and A/H3N2 have been used effectively for more than 20 years for controlling and preventing potential pandemics. However, influenza vaccines against the B/Victoria and B/Yamagata lineages were used interchangeably. In the 2016–2018 seasons, the trivalent influenza vaccine did not include the B/Yamagata lineage.

A high incidence of influenza-like illnesses was reported in January 2018 by the China CDC^1^. To investigate whether this was due to the unavailability of B/Yamagata vaccine, we collected 9914 nasal swabs from patients presenting with influenza-like symptoms in Guangzhou, Southern China from January through March in 2018. Most of the patients were outpatients (88.7%). Influenza-positive samples were further subtyped by sequencing and phylogenetic analysis of HA and NA genes. The data provide a view into the molecular epidemiology of the influenza viruses circulating in the current population, which may, in turn, confirm the efficacy of the influenza vaccines administered as well as provide insights for the future development and deployment of effective subtype or lineage-specific influenza vaccines.

## Materials and Methods

### Influenza Diagnostic Tests

From January through March 2018, nasal swab specimens from 9914 patients with influenza-like symptoms were collected and tested for influenza viral antigens (Li et al., 2019) using the Clearview^®^ Exact Influenza A&B kit with the colloidal gold detection method (Jennings et al., 2009)^2^. Briefly, each nasal swab was eluted with 192 μL lysis buffer and the immunochromatographic strip in the kit was inserted into the eluent and incubated for 15 min at room temperature. Then the results were determined according to the manufacturer’s instructions. The interaction between the colloidal gold conjugated antibodies and the influenza antigens will indicate red in the test line. This study was approved by the Institutional Review Board of the Affiliated Cancer Hospital of Guangzhou Medical University in accordance with the Declaration of Helsinki, with the patient consent for using left-over specimens waived.

### RNA Extraction, RT-PCR and Sequencing

Each nasal swab was eluted with 1 mL DMEM. The eluent was immediately stored at −80°C. Subsequently, 1 mL Trizol was used to extract viral RNA from the 200 μL of nasal swab eluent, and 200 μL chloroform was added immediately and centrifuged at 12,000 × g. The upper aqueous layer was transferred to a tube, with 1 mL 100% isopropanol for RNA precipitation. Following centrifuge at 12,000 × g, 1 mL of 75% cold ethanol to the pellet as a wash. The RNA was eluted into 20 μL RNase-free water. This RNA was reverse transcribed into cDNA using the PrimerScript^TM^ RT Reagent Kit with gDNA Eraser (TAKARA), according to the manufacturer’s protocol. All primers used in this experiment are based on the “WHO protocols for molecular diagnosis of influenza virus”^3^ (Table 1). The cDNA was amplified using the Premix Taq^TM^ (TaKaRa^TM^ Taq Version 2.0), under the following conditions: Initial denaturation at 94°C for 2 min; amplification in 40 cycles of 94°C for 30 s/50°C for 30 s/72°C for 2 min; and final extension for 10 min at 72°C. These amplified sequences were confirmed by Sanger DNA sequencing using the primers listed in Table 1 as sequencing primers.

**TABLE 1:** Primers used for RT and PCR amplification of influenza A and B viruses.

Purpose	Primer name	Sequence (5′–3′)	Expected size
RT for influenza A	uni12W	AGCRAAAGCAGG	N/A
RT for influenza B	Buni11W	AGCAGAAGCGS	N/A
PCR for the HA gene of influenza A	H1F1	AGCAAAAGCAGGGGAAAATAAAAGC	1.7 kb
	H3A1F6	AAGCAGGGGATAATTCTATTAACC	
	H5A1F1	AGCAAAAGCAGGGGTATAATC	
	HARUc	ATATCGTCTCGTATTAGTAGAAACAAGGGTGTTTT	
PCR for the NA gene of influenza A	N1F1	AGCAAAAGCAGGAGTTTAAAATG	1.3 kb
	NARUc	AGTAGAAACAAGGAGTTTTTT	
PCR for the HA gene of influenza B	BHAF1u^a^	AGCAGAAGCAGAGCATTTTCTAATATC	1.4 kb
	BHAR1341	TTCGTTGTGGAGTTCATCCAT	
nested-PCR for the HA gene of influenza B	Bvf224	ACATACCCTCGGCAAGAGTTTC	Victoria lineage:
	Bvr507	TGCTGTTTTGTTGTTGTCGTTTT	284 bp
	BYf226	ACACCTTCTGCGAAAGCTTCA	Yamagata lineage:
	BYr613	CATAGAGGTTCTTCATTTGGGTTT	388 bp

**Primers were chosen from the “WHO protocols for molecular diagnosis of influenza virus.” ^*a*^Modified from primer sequences WHO recommended.**

### Phylogenetic Sequence Analyses

Phylogenetic analysis was performed by Molecular Evolutionary Genetics Analysis (MEGA X)^4^ (Kumar et al., 2018). The phylogenetic tree was generated by the maximum-likelihood method with 1,000 bootstrap replicates using the MEGA X by applying default parameters (Zhang et al., 2017, 2019). Sequences of HA and NA genes were retrieved from Influenza Research Database^5^ are used for comparison. The gene reference sequences of H1N1, H3N2, and Victoria and Yamagata lineages were extracted from published sequences, using BLAST, obtained from strains that circulated worldwide in recent years, as well as China strains and northern hemisphere vaccine strains recommended by WHO and the reference strains of known clades as reported by WHO.

### Amino Acid Mutation Analysis

Amino acid mutations in the HA genes of 23 influenza A/H1N1 isolates from residues 7 to 566 were compared to homologous sequences in the 2017–2019 vaccine strain (A/Michigan/45/2015). In addition, amino acid mutations of the HA genes of 80 influenza B/Yamagata isolates from residues 96–215 were compared to the equivalent sequences from the 2018–2019 vaccine strain (B/Phuket/3073/2013). Amino acid mutations in the HA genes from the 19 influenza B/Victoria isolates and the 2018–2020 B/Victoria vaccine strain from residues 93 to 180 were also compared to the counterparts in the 2016–2018 northern hemisphere vaccine strain (B/Brisbane/60/2008). These analyses were compared using the software CLUSTAL.

### 3D Protein Structure Analysis

The SWISS-MODEL server and web-tool^6^ (Waterhouse et al., 2018) was used to predict the 3D structure of the HA protein of the two northern hemisphere vaccine strains (A/Michigan/45/2015 and B/Phuket/3073/2013). SWISS-MODEL searches for related protein structure templates from the SMTL database by BLAST and HHblits were conducted to ensure accuracy and sensitivity as much as possible. The crystal structure of the B/Brisbane/60/2008 vaccine strain of the HA protein was retrieved from NCBI. The PDB ID: 4FQM. PyMOL software^7^ as used to overlay the positions of the antigenic sites and mutation sites on the HA crystal structure of influenza A and B isolates.

### HA and NA Gene Sequences Annotation and GenBank Accession Numbers

Sequence data were assembled with the SeqMan Pro software 7.0.1 (DNASTAR, Inc., Madison, WI, United States). Nucleotide and amino acid sequences of the HA and NA genes were aligned using the CLUSTAL and BLAST software. These HA and NA gene sequences from influenza A and B were archived in GenBank with the following accession numbers: (1) MN653601-MN653624 (influenza A, HA gene); (2) MN653549-MN653572 (influenza A, NA gene); and (3) MN653573-MN653600 and MT123908-MT123978 (influenza B, HA gene).

## Results and Discussion

### The Epidemiology of Influenza A and B Infections

Type-specific colloidal gold test revealed that 2547 of 9914 patients (25.7%) during this outbreak were positive for influenza viruses, of which 312 (3.1%) were identified as influenza A and 2241 (22.6%) as influenza B. The dominant type circulating was influenza B (87.8%). Outpatients of influenza A and B positive accounted for 297 (95.2%) and 2141 (95.5%), respectively. Positive specimens collected during the outbreak were selected randomly (100 specimens for each month) for further subtyping by Sanger sequencing DNA sequencing of the HA and NA genes. The sequence data resulted in the typing of the influenza A strains as 23 H1N1 with one H3N2, and the typing of influenza B as identified 80 Yamagata and 19 Victoria strains.

**FIGURE 1:**
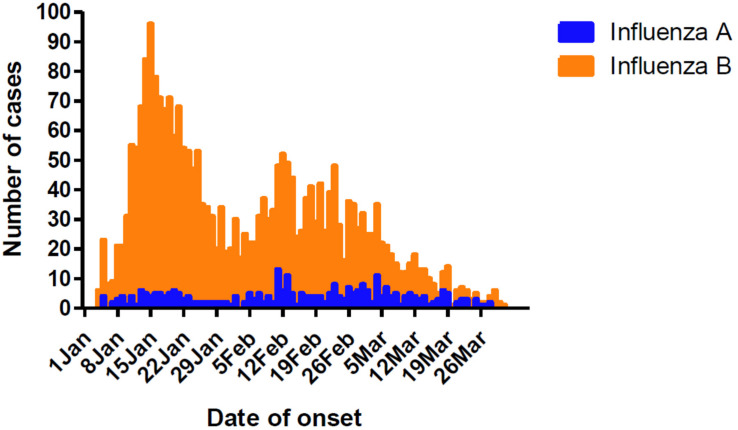
Timeline and type distribution of influenza A and B cases of onset in Guangzhou, Southern China, January–March 2018. The number of cases of each influenza A and B are shown encompassing the two peaks of the epidemic.

Influenza cases peaked initially from January 8 to 21, followed by another peak from February 5 to March 4 (Figure 1). This was different from the Northern China epidemic profile (one peak, January 1–21). During the two peaks, there was a total of 1776 influenza positive cases. Among these, influenza B accounted for 1579 cases, with only 203 as influenza A positive. These include six cases testing as both influenza A and B positive.

During the outbreak, influenza A and B viruses circulated in all age groups. Median ages of the patients were 12 and 17, respectively, with the male to female ratio of 1.1:1. The highest positive rate of influenza A and B infections appeared in children between the ages of 5–14 years, with incidences of 4.2 and 31.7%, respectively (Table 2). All age groups were susceptible to influenza B virus, accounting for 17.9–31.7% of the flu-like cases.

**TABLE 2:** The age distribution of influenza A and B patients in Guangzhou, Southern China, January–March 2018.

	Cases	Influenza	Influenza	Influenza
Age	detected	A (%)	B (%)	A and B (%)
0–4	3307	117 (3.5)	671 (20.3)	787 (23.8)
5–14	1274	53 (4.2)	404 (31.7)	456 (35.8)
15–24	958	27 (2.8)	258 (26.9)	284 (29.6)
25–59	3098	91 (2.9)	679 (21.9)	769 (24.8)
≥60	1277	24 (1.9)	229 (17.9)	251 (19.7)
Total	9914	312 (3.1)	2241 (22.6)	2547 (25.7)

### The Subtype Distribution and Phylogenetic Analysis of Influenza A and B Viruses

**FIGURE 2:**
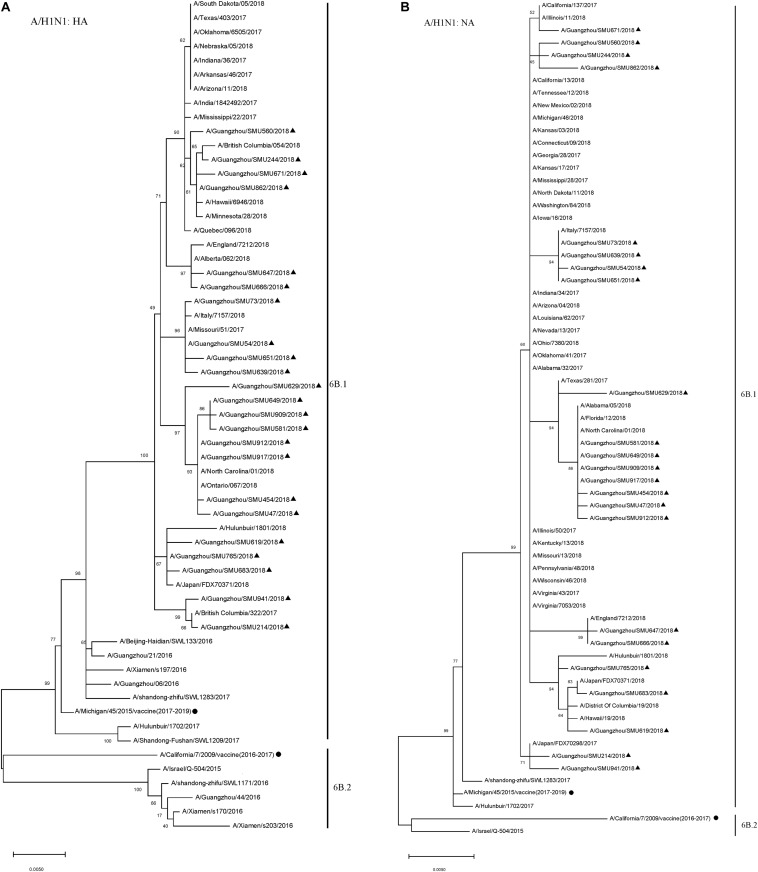
Phylogenetic analysis of the HA **(A)** and NA **(B)** genes from influenza A/H1N1 viruses. The phylogenetic tree was generated by the maximum-likelihood method with 1,000 bootstrap replicates using the MEGA X by applying default parameters. Bootstrap numbers shown at the nodes mean the percentages of 1000 replications producing the clade. The scale bar indicates the units of nucleotide substitutions per site. Nucleotide sequences of HA and NA genes retrieved from Influenza Research Database (https://www.fludb.org/brc/influenza_sequence_search_segment_display.spg?method=ShowCleanSearch&decorator=influenza) are used as references. Sequences of the isolates obtained in this study are noted (▲), as well as sequences of the northern hemisphere vaccine strains of known clades as reported by WHO as reference (•).

To reveal the subtypes circulating during this outbreak, the HA and NA genes were further analyzed using their sequences. Phylogenetic analysis showed that the HA and NA genes of the 23 H1N1 isolates had close sequence relationships to the strains circulating in Northern China, Japan, Italy, England, and the USA during 2017–2018, as well as to the 2016–2017 vaccine strain (A/California/7/2009/vaccine) and 2017–2019 vaccine strain (A/Michigan/45/2015/vaccine). The phylogenetic analysis indicated that there were few HA and NA genetic variations between the A/H1N1 isolates circulating in 2018 and the 2016–2019 vaccine strains. All of the A/H1N1 strains circulating during January to March, as well as the 2017–2019 vaccine strain (A/Michigan/45/2015), fell into clade 6B.1, which is defined by the HA1 amino acid substitutions S84N, S162N and I216T. The 2016–2017 vaccine strain (A/California/7/2009) and the other four China strains circulating in 2016 formed a clade noted as 6B.2 (Figure 2).

**FIGURE 3:**
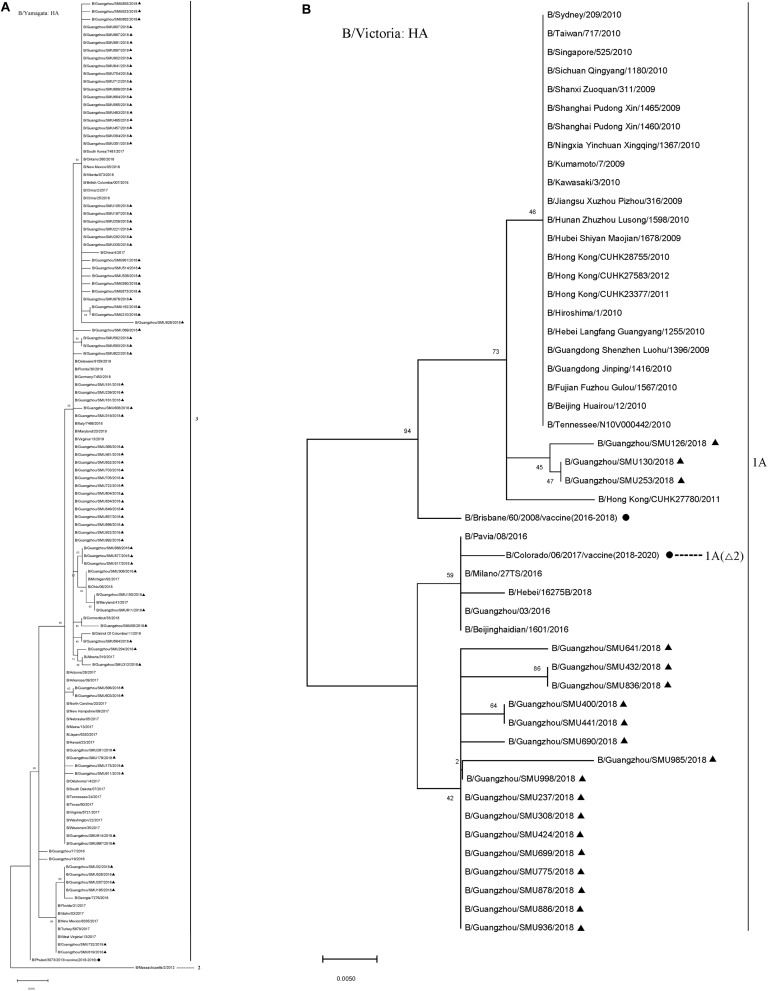
Phylogenetic analysis of the HA genes of influenza B/Yamagata isolates **(A)** and B/Victoria isolates **(B)**. The phylogenetic trees were generated by the maximum-likelihood method with 1,000 bootstrap replicates using the MEGA X by applying default parameters. Bootstrap numbers shown at the nodes mean the percentages of 1000 replications producing the clade. The scale bar indicates the units of nucleotide substitutions per site Nucleotide sequences of the HA genes retrieved from the Influenza Research Database (https://www.fludb.org/brc/influenza_sequence_search_segment_display.spg?method = ShowCleanSearch& decorator = influenza) are used as references. Sequences of the isolates obtained in this study are noted (▲), as well as sequences of the northern hemisphere vaccine strains of known clades as reported by WHO as reference (•). Δ2 means two amino acid deletions (177K and 178N).

The majority (80/99) of the influenza B isolates belonged to the Yamagata lineage, which was further divided into two sub-branches: One was phylogenetically close to strains circulating in China, Japan, Italy, South Korea, German, Canada, and the USA during 2016–2018 and the other was close to strains B/Georgia/7276/2018, B/Florida/31/2017, B/Idaho/03/2017, B/New Mexico/6335/2017, B/Turkey/5879/2017, and B/West Virginia/13/2017 (Figure 3). Influenza B Yamagata-lineage was separated previously into two major antigenically distinct clades (clades 2 and 3), based on phylogenetic analysis of its HA and NA genes (Barr et al., 2010, 2014). All of the B/Yamagata strains circulating during January to March and the 2018–2019 vaccine strain (B/Phuket/3073/2013) parsed into clade 3.

During the 2016–2018 seasons, the trivalent vaccine didn’t include B/Yamagata lineage, so its effectiveness could not be determined. However, it may be speculated that the absence of B/Yamagata vaccine might have contributed to an influenza B outbreak due to the low herd immunity.

The 19 B/Victoria strains clustered within two close sub-branches, 16 of which were close phylogenetically to the 2018–2020 vaccine strain (B/Colorado/06/2017) and three of which were close to the 2016–2018 vaccine strain (B/Brisbane/60/2008) (Figure 3). All of the 19 B/Victoria strains were members of clade 1A.

### Amino Acid Mutations and 3D Structure Analysis of the HA Antigenic Sites Indicate the Potential Effect on Vaccines

Current influenza vaccines provide important protection in humans by inducing strain-specific neutralizing antibodies targeting highly variable antigenic epitopes in the globular domain of the HA protein that is in the virus envelope. They play a critical role in host cell recognition, viral binding, as well as the subsequent fusion and entry processes (Shen et al., 2013; Qi et al., 2018). Amino acid mutations of the HA genes of circulating influenza isolates and the predicted 3D structure were further analyzed, and compared to the northern hemisphere vaccine strains.

**FIGURE 4:**
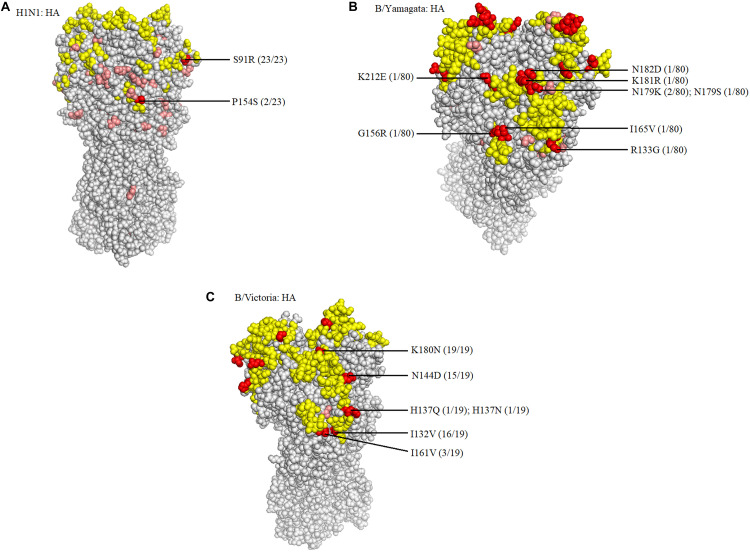
Structural modeling of the HA mutations. The hemagglutinin structures of influenza A/H1N1 **(A)**, B/Yamagata **(B)**, and B/Victoria **(C)** vaccine strains are presented, in which the antigenic epitopes of the protein are mapped and marked in yellow. Amino acid mutations of the isolates are either marked in pink, if not located in antigenic sites, or in red, if located in antigenic sites. The frequency was also noted.

When compared with the A/H1N1 2017–2019 vaccine strain (A/Michigan/45/2015), there were three major amino acid mutations within the HA gene of all 23 isolates (S91R, S181T, and I312V), of which one mutation, S91R, located in the Cb antigenic site was identified in all 23 isolates (Figure 4A). Another mutation P154S in the Ca2 antigenic site was also found in two isolates (SMU214 and SMU941). Except for these mutations, no additional mutations were located within these antigenic sites of HA gene: Sa, Sb, Ca1, Ca2, Cb (Huang et al., 2013; Matsuzaki et al., 2014). S181T was identified in all the 23 isolates, although the meaning of the mutation is unknown (Table 3). To be clear, it is not known if these patients were vaccinated for influenza. Whether these mutations actually changed the immunogenicity of these isolates needs to be investigated further. However, the incidence of influenza A was significantly lower than influenza B in Jan-Mar 2018. The use of the influenza A vaccine appears to have been effective in controlling the potential influenza A outbreak.

**TABLE 3:** Amino acid mutations in the HA genes of A/H1N1 isolates.

A/H1N1 strain						Cb		Ca2														
Positions (frequency)	10 (2)	17 (1)	62 (1)	83 (1)	89 (1)	91 (23)	130 (1)	154 (2)	155 (4)	163 (1)	181 (23)	200 (6)	230 (1)	250 (2)	273 (4)	284 (2)	289 (4)	299 (1)	312 (23)	389 (2)	496 (1)	513 (1)
**A/Michigan/45/2015/vaccine (2017–2019)**	**Y**	**A**	**R**	**E**	**T**	**S**	**R**	**P**	**H**	**K**	**S**	**S**	**E**	**L**	**T**	**I**	**V**	**P**	**I**	**I**	**V**	**N**
SMU683					S	R					T								V			
SMU619						R				T	T								V			
SMU581						R					T				K				V			
SMU244/671/862 (3)						R					T	P			I				V			
SMU560						R					T	P							V			H
SMU666	C					R					T	P		I					V		I	
SMU647	C	T				R					T	P		I					V			
SMU214						R		S			T		D			T			V	L		
SMU941			K			R		S			T					T		S	V	L		
SMU73						R	K		Y		T						A		V			
SMU639				D		R			Y		T						A		V			
SMU651/54 (2)						R			Y		T						A		V			
SMU47/454/629/649/765/909/912/917 (8)						R					T								V			

*Twenty-three isolates were included for analysis. The northern hemisphere 2017–2019 vaccine strain (A/Michigan/45/2015) was used as reference. Amino acids highlighted in red indicate the mutations in the antigenic sites. Bold letterings represent amino acids comprising the HA protein of the vaccine strain. The HA antigenic epitopes of Cb are located at the amino acid residues 87–88, 90–92, and 132; Ca2 is located at the amino acid residues 154, 157, 159, and 238–239.*

Because there was no influenza B/Yamagata vaccine used during the 2017–2018 season, the 2018–2019 influenza B/Yamagata vaccine strain (B/Phuket/3073/2013) was used as reference. The HA gene of most of the isolates was conserved. Only five out of 80 isolates contained one non-synonymous substitution, located in the 120-loop, 150-loop, 160-loop, and 190-helix sites (Wang et al., 2008), respectively (marked in red in Table 4). One isolate (SMU926) harbored four mutations in the 160-loop site. Except for these mutations, no other changes were found in the HA antigenic sites (Table 4). The mutation L187Q was noted in all 80 isolates, but it was not found in any antigenic sites and therefore may not affect the antigenicity. At amino acid residue 179, three of eighty isolates had amino acid mutations in the antigenic epitopes (Figure 4B). For the other six mutations, they were only found in one isolate for each mutation. These indicated that the 2018–2019 B/Yamagata vaccine strain was antigen-specific and appropriate for the 2018 circulating B/Yamagata strains.

**TABLE 4:** Amino acid mutations in the HA genes of B/Yamagata isolates.

B/Yamagata strain				120-loop	150-loop			160-loop					190-helix
Positions (frequency)	103 (1)	127 (1)	129 (1)	133 (1)	156 (1)	165 (1)	169 (1)	179 (3)	181 (1)	182 (1)	187 (80)	199 (1)	212 (1)
**B/Phuket/3073/2013/vaccine (2018–2019)**	**R**	**R**	**Y**	**R**	**G**	**I**	**A**	**N**	**K**	**N**	**L**	**E**	**K**
SMU06/294 (2)								K			Q		
SMU926			C	G				S	R	D	Q		
SMU981					R						Q		
SMU882						V					Q		
SMU622											Q		E
SMU369	K										Q		
SMU536											Q	D	
SMU806							S				Q		
SMU911		G									Q		
The other 70 isolates											Q		

*Eighty isolates were included for analysis. The northern hemisphere 2018–2019 vaccine strain (B/Phuket/3073/2013) was used as reference. Amino acids highlighted in red indicate the mutations in the antigenic sites. Amino acids residues of the vaccine strain are in bold. For reference, the antigenic epitopes of the HA protein are located at the amino acid residues 131–152 (120-loop); 156–165 (150-loop); 177–186 (160-loop); and 211–219 (190-helix).*

When compared with the B/Victoria 2016–2018 vaccine strain (B/Brisbane/60/2008), all 19 isolates had one common amino acid mutation (K180N), located in the HA 160-loop (Table 5). Five mutations located in the HA antigenic epitopes were identified in the 19 B/Victoria isolates: K180N (19/19), I132V (16/19), N144D (15/19), I161V (3/19), H137Q or H137N (1/19), respectively (Figure 4C). Note that the mutations K180N, I132V, and N144D occurred in most of the isolates. In the 150-loop, three isolates contained a mutation I161V. Additionally, 17 of 19 isolates had three mutations located in the three antigenic sites (120-loop, 150-loop, and 160-loop). When compared with the B/Victoria 2018–2020 vaccine strain (B/Colorado/06/2017), surprisingly, all 19 isolates had K180N mutation in the 160-loop and 15 of 19 isolates had a G144D mutation in the 120-loop. A previous study found that the amino acid substitution N180K altered the epitope and affected the viral antigenicity. Human antibodies did not substantially inhibit the hemagglutination reaction in the hemagglutination inhibition tests (Nakagawa et al., 2005). Such variants may be important in future epidemics. Except for the B/Colorado/06/2017/vaccine strain, which had two deletions (Δ2) in the HA antigenic sites (177K and 178N), the other isolates had no amino acid deletions at any of the antigenic sites (Table 5). A larger than expected number of mutations found within HA multiple antigenic epitopes of B/Victoria isolates suggest that the HA gene may be more variable than previously thought (Lindstrom et al., 1999). The isolates accumulated mutations at several antigenic sites, which may reduce the protective efficacy of the vaccine. Therefore, the B/Vitoria vaccine strain used in 2018–2020 might not be appropriately selected for Southern China should this be the case. According to the report of the National Health Commission of China, the B/Victoria strain re-dominated in China in 2019^8^. In the context of our study, this indicates that the protective efficacy of 2018–2019 B/Victoria vaccine is likely reduced due to the mutations in the antigenic sites of the HA protein, particularly if the vaccination rate in China remained at the same level in 2019 as in prior years.

**TABLE 5:** Amino acid mutations in the HA genes of B/Victoria isolates.

B/Victoria strain	120-loop			150-loop		160-loop		
Positions (frequency)	132 (16)	137 (2)	144 (15)	161 (3)	169 (1)	177 (0)	178 (0)	180 (19)
**B/Brisbane/60/2008/vaccine (2016–2018)**	**I**	**H**	**N**	**I**	**A**	**K**	**N**	**K**
B/Colorado/06/2017/vaccine (2018–2020)	V		G			deletion	deletion	
SMU130/253 (2)				V				N
SMU126		Q		V				N
SMU690	V	N						N
SMU985	V		D		T			N
SMU237/308/400/424/441/432/836/998/641/699/775/878/886/936 (14)	V		D					N

*Nineteen isolates were included for analysis. The northern hemisphere 2016–2018 vaccine strain (B/Brisbane/60/2008) was used as reference. The northern hemisphere 2018–2020 vaccine strain (B/Colorado/06/2017) was also used for comparison. The amino acids in red indicate the mutations in the antigenic sites. Amino acids comprising the vaccine strain are in bold. The antigenic epitopes of the HA protein are located at amino acid residues 131–152 (120-loop); 156–165 (150-loop); and 177–185 (160-loop).*

## Conclusion

According to the report of the National Health Commission of China, from January through March of 2018, 487,773 people were infected with influenza, of which 119 died. During the outbreak, both influenza A and B dominated in Northern China. In contrast, our study found that influenza B, rather than influenza A, dominated in Southern China. Throughout the years, influenza A viruses have attracted a great deal of attention and have caused several global pandemics due to their strong transmission capacity and great variability. In contrast, there is less literature focused on the epidemiology and societal burdens of influenza B, particularly outside the United States and Europe (Caini et al., 2015). Nevertheless, influenza B remains an critical respiratory pathogen that imparts an important and large public health impact (Paul Glezen et al., 2013), and studies such as this provide important context for both influenza A and B, as well as insights into the dynamics of their epidemiology. It should be noted that a domination of influenza B/Yamagata in both Northern and Southern China has occurred twice, in the past 2007–2008 and in 2014–2015 (Yu et al., 2013; Yang et al., 2018). In this study, we found that, 3 years later, influenza B/Yamagata dominated once again in Southern China.

The highest incidence of influenza infection occurred among the 5–14-year-old children, which indicates that children and adolescents were more susceptible to influenza viruses. Outpatients accounted for more than 95% of influenza cases, which suggested that the illness was not particularly severe during this outbreak. H1N1 was the major subtype of influenza A (95.8%) and the Yamagata lineage was the major lineage of influenza B (80.8%). The outbreak of influenza viruses in Southern China in 2018 was mainly caused by Yamagata lineage, which was phylogenetically close to other influenza B strains circulating worldwide in the same timeframe.

With respect to the phylogenetic, mutation, and 3D structure analysis of HA genes, the A/H1N1 vaccine strain used in 2017–2018 was antigen-specific and appeared to be effective in this season. However, the low influenza vaccination rate in China might have contributed to the emergence of H1N1 cases^9^. Due to the lack of the influenza B/Yamagata vaccine during 2016–2018, the outbreak of the influenza B/Yamagata virus in 2018 might have been expected. According to our analysis, the influenza B/Yamagata vaccine strain used during the subsequent season (2018–2019) was antigen-specific for the circulating strains. An outbreak of influenza B/Yamagata infections did not occur in 2019 perhaps due to the use of the inactivated B/Yamagata vaccine. However, the HA gene of the 2018 B/Victoria isolates had accumulated mutations in several antigenic sites, which may have potentially changed the immunogenicity. Furthermore, when compared with the B/Vitoria 2018–2019 vaccine strain, more HA mutations were found in the circulating isolates, which may have reduced the protective efficacy of B/Vitoria 2018–2019 vaccine. This is an indication that the vaccine strain might not have been the best candidate vaccine for Southern China in future influenza seasons. According to the reports of the United States CDC, although the influenza vaccination rate for adults was as high as 45.3% in 2018–2019, an increase of 8.2% from the 2017–2018 influenza season, a severe influenza infection still broke out in 2019–2020, causing more than 10,000 deaths, primarily caused by influenza B/Victoria lineage (Owusu et al., 2020)^10^. HA gene variations may and should be considered for evaluating potential vaccine efficacy.

Previous studies demonstrated that vaccination with the live and attenuated influenza vaccine elicited lung CD4^+^ and virus-specific CD8^+^ T cell responses, similar in phenotype to those generated by influenza virus infection, and ultimately established lung Tissue-resident memory T cells (TRM) capable of providing long-term, hetero-subtypic protection to multiple, non-vaccine influenza strains. In contrast, vaccination with inactivated influenza vaccine generated durable, strain-specific humoral immunity but failed to elicit T cell responses (Zens et al., 2016). In China, all the influenza vaccines approved and used are inactivated vaccines. Therefore, the effect of mutations on HA T cell epitopes was not analyzed.

The epidemiological, phylogenetic, mutation, and 3D structural analyses presented in this study, contribute to a better understanding of circulating influenza strains by revealing critical mutations, allowing for an evaluation of vaccine efficacy, and providing a basis for the improved selection of more specific and effective influenza vaccine candidate strains. A continuing surveillance of the sequence variations of the HA genes is important for managing, controlling, and limiting future influenza outbreaks and pandemics. These surveys and characterizations of circulating influenza strains are especially important should there be emergent or re-emergent influenza viral pathogens crossing the human-animal interface.

## Data Availability Statement

All datasets generated for this study are included in the article.

## Author Contributions

QZ, YY, and ZY conceived and designed the experiments. ZY collected and provided clinical samples. YY performed the experiments. YY, JO, SZ, KM, WL, WG, XW, JZ, and QZ analyzed the data. BZ, WZ, CW, WS, JW, DS, ZY, and QZ contributed to the preparation of the manuscript.

## Conflict of Interest

The authors declare that the research was conducted in the absence of any commercial or financial relationships that could be construed as a potential conflict of interest.

## Footnotes

1 http://www.chinacdc.cn/jkzt/crb/bl/lxxgm/zstd/201801/t20180108_158017.html

2 https://www.alere.com/zh/zh/product-details/clearview-exact-influenza-a-and-b.html

3 https://www.who.int/influenza/gisrs_laboratory/molecular_diagnosis/en

4 http://www.megasoftware.net/index.html

5 https://www.fludb.org/brc/influenza_sequence_search_segment_display.spg?method=ShowCleanSearch&decorator=influenza

6 https://swissmodel.expasy.org/

7 https://pymol.org/2/

8 http://www.chinaivdc.cn/cnic/zyzx/lgzb/

9 http://www.chinacdc.cn/mtbd_8067/201810/t20181030_196735.html

10 https://www.cdc.gov/flu/fluvaxview/coverage-1819estimates.htm
